# An Ethics Framework for Big Data in Health and Research

**DOI:** 10.1007/s41649-019-00099-x

**Published:** 2019-10-01

**Authors:** Vicki Xafis, G. Owen Schaefer, Markus K. Labude, Iain Brassington, Angela Ballantyne, Hannah Yeefen Lim, Wendy Lipworth, Tamra Lysaght, Cameron Stewart, Shirley Sun, Graeme T. Laurie, E Shyong Tai

**Affiliations:** 1 Centre for Biomedical Ethics, Yong Loo Lin School of Medicine, National University of Singapore, Singapore; 2 Centre for Social Ethics and Policy, School of Law, University of Manchester, Manchester, UK; 3 Department of Primary Health Care & General Practice, University of Otago, Dunedin, New Zealand; 4 Division of Business Law, College of Business, Nanyang Technological University, Singapore; 5 Sydney Health Ethics, Faculty of Medicine and Health, The University of Sydney, Sydney, Australia; 6 The University of Sydney Law School, Sydney, Australia; 7 School of Social Sciences, College of Humanities, Arts, & Social Sciences, Nanyang Technological University, Singapore; 8 School of Law and JK Mason Institute for Medicine, Life Sciences and the Law, University of Edinburgh, Edinburgh, UK; 9 Saw Swee Hock School of Public Health, National University of Singapore, Singapore; 10 Division of Endocrinology, National University Hospital, Singapore

**Keywords:** Ethics framework, Health and research, Open sharing, Data repositories, Precision medicine, Real-world evidence, Artificial intelligence, Public-private partnership, Cross-sectorial data

## Abstract

Ethical decision-making frameworks assist in identifying the issues at stake in a particular setting and thinking through, in a methodical manner, the ethical issues that require consideration as well as the values that need to be considered and promoted. Decisions made about the use, sharing, and re-use of big data are complex and laden with values. This paper sets out an *Ethics Framework for Big Data in Health and Research* developed by a working group convened by the *Science, Health and Policy-relevant Ethics in Singapore* (SHAPES) *Initiative.* It presents the aim and rationale for this framework supported by the underlying ethical concerns that relate to all health and research contexts. It also describes a set of substantive and procedural values that can be weighed up in addressing these concerns, and a step-by-step process for identifying, considering, and resolving the ethical issues arising from big data uses in health and research. This Framework is subsequently applied in the papers published in this Special Issue. These papers each address one of six domains where big data is currently employed: openness in big data and data repositories, precision medicine and big data, real-world data to generate evidence about healthcare interventions, AI-assisted decision-making in healthcare, public-private partnerships in healthcare and research, and cross-sectoral big data.

## Background

The use, sharing, and re-use of big data is a defining feature of the current health and research landscape. A number of technological developments, such as artificial intelligence, to give one example, can only advance with the use of big data. While much has been written about the ethics of big data in a variety of contexts, there is little guidance around which values are at stake and how we should make decisions in an increasingly complex health and research environment. This paper proposes an *Ethics Framework for Big Data in Health and Research* (hereafter Framework) and provides insight into the values that are often central to decisions made in a number of contexts where big data is used. The development of the Framework has arisen from an international collaborative effort, a working group convened by the *Science*, *Health and Policy-relevant Ethics in Singapore* (SHAPES) *Initiative*. Throughout the drafting process of the Framework, the international working group (SHAPES Working Group) has benefitted from input from a number of experts in Singapore and around the world.

## Definitions and Scope of the Framework

### The Nature of ‘Big Data’

Big data is increasingly used in many different sectors from financial services, to security, and law enforcement. Our work focuses on how big data is used in health and research contexts. Some of these contexts will look familiar to those in public health and epidemiology, as they handle very large data sets from a variety of sources and sometimes in real time. However, big data uses are going well beyond these traditional contexts, especially with the emergence of immense online repositories and technologies, such as artificial intelligence (as discussed in this Special Issue), transforming how data is stored, accessed, and shared in health and research contexts.

The term ‘big data’ has been defined by a number of scholars, practitioners, and policymakers in various ways but there are key characteristics of big data that exist across these different accounts. We will not attempt to provide our own definition of ‘big data’ here; we will focus instead on key characteristics frequently associated with the term, all of which point to its complexity (Baro et al. 2015). These characteristics have been articulated in terms of the following:

- ‘3 Vs’ (volume, variety, velocity);
- ‘4 Vs’ (volume, variety, velocity, variability);
- ‘6 Vs’ (volume, variety, velocity, variability, veracity, value); and
- ‘7 Vs’ (volume, variety, velocity, variability, veracity, value, visualisation) (Sivarajah et al. 2017).

In this work, we have focused on core characteristics considered by all, i.e. volume, variety, and velocity (Box 1). In subsequent sections, it will become clear how these characteristics raise particular ethical challenges when big data is generated, used, and shared in the context of health and research.

**Box 1:** Key characteristics of ‘big data’

Volume: The sheer quantity of data, taking into account the number of persons whose data is contained in given datasets and the level of detail about each individual. Variety: The substantial diversity of data forms about individuals (e.g. structured, unstructured, images, audio) as well as the diversity of sources for that data (e.g. scientific data, user-generated data, web data). Velocity: The great speed at which data can be transmitted and analysed.	

These characteristics go beyond how given datasets are being used at present and point towards how they may potentially be used in the future. That potential, even if not presently realised, is ethically relevant insofar as it raises questions about how to manage and govern big data and data that may potentially become big data. Such considerations will enable the eventual realisation of that great potential to occur in a responsible and ethically defensible manner.

The foreseeable benefits of exploiting big data in health and research are varied and hold the promise of being transformative. In the healthcare sector alone, the benefits range from infrastructural, operational, organisational, managerial, and strategic which ultimately translate into improved treatments, more efficient service delivery, and cost savings for healthcare organisations (Wang et al. 2018). An example of operational benefits that would impact on patients directly is the improvement of the quality and accuracy of clinical decisions (Wang et al. 2018).

While the potential arising from big data in all fields, including health and research, is widely recognised, so too are the numerous challenges that big data poses. These challenges have been identified as relating to the characteristics of big data (*data challenges*); issues around capturing, integrating, transforming, analysing, and interpreting big data (*process challenges*); and around addressing privacy concerns, data security, governance, and data sharing, as well as operational and ownership issues (*management challenges*) (Sivarajah et al. 2017). The Framework we have developed primarily focuses on *management challenges* but we acknowledge that these sets of challenges inevitably influence each other*.*

### Defining ‘Health’ and ‘Research’

It is useful to make clear which specific contexts our Framework focuses on by providing definitions for *health* and *research*. By ‘health’, we mean systems or fields whose primary aim is the maintenance or restoration of our physical and mental condition and wellbeing. This extends to clinical medicine, epidemiology, and public health. When we talk about ‘research’, we refer to any systematic investigation with the intention of generating or contributing to generalisable knowledge. Specifically, we will focus on research activities that are of relevance to health, understood to also extend to wellbeing and welfare. Even though we provide discrete definitions, we acknowledge the blurring of boundaries between research and treatment (Kass et al. 2013), which impacts on our definition of ‘health’.

### What is an Ethical Decision-making Framework?

This Framework is a tool for deliberating about issues related to big data by bringing to the fore relevant values which guide or ‘frame’ decision-making (Dawson 2010). The Framework is neither a theory that helps justify actions^1^ nor a model that provides a simplified means of understanding a complex issue, although it can be linked to theories and explanations embedded within it (Dawson 2010). Characteristics of a good decision-making framework, as described in (Dawson 2010), include the following:

- It articulates its aim and scope
- It is practical
- It makes the values at stake explicit. As a starting point, the values are considered to be of equal weight (i.e. no value always has greater priority than another) and they guide decision-making^2^
- It is flexible, i.e. it does not force any particular kind of decision
- It is problem-driven rather than theory-driven, i.e. it is intended to address real-world dilemmas and quandaries rather than to explore theoretical positions
- It provides explicit guidance on what kinds of issues to consider through a structured series of questions.

Points to consider regarding the use of frameworks include:

- Frameworks do not provide the answer to particular questions; rather, they help us think through the issue(s) and arrive at an answer, which may, in fact, require further consideration.
- If there are underlying assumptions that implicitly give priority to a value, a framework can reinforce certain positions rather than being genuinely exploratory of all relevant values (Dawson 2010; Grill and Dawson 2017). This distinguishes ethical decision-making frameworks from legislative and regulatory frameworks, which prioritise certain values and articulate resulting obligations.
- Ethical decision-making frameworks often allude to or incorporate a deliberative balancing process to help work-through tensions between conflicting values.

It is important to remember that the use of frameworks still requires personal and general wisdom in decision-making. Personal and general wisdom incorporate features such as deep insight, sound judgement, acknowledgment, and tolerance of uncertainty, as well as a balanced outlook on solutions (Staudinger 2013; Staudinger and Glück 2011). Deliberation of this kind also requires the exercise of personal discretion.

## Rationale, Aims, and Audience

### The Need for an Ethics Framework for Big Data in Health and Research

Several complex ethical issues arise in considering and making decisions about uses of big data. Although some of these issues are also present in more conventional data ecosystems, they are either more acute in the context of big data, and/or traditional means of addressing these issues are no longer fit for purpose. In the subsequent two sections, we articulate what we take to be key issues.

### Inability to Rely Solely on Data Masking Techniques and De-identification

The issue of anonymisation^3^ has become highly technical and care needs to be taken when making claims about the associated risks or lack thereof, especially because of rapid developments in data science and the different thresholds for considering data ‘anonymised’ given the various techniques available. In many jurisdictions, there remains a bright regulatory line between ‘identifiable^4^’ and ‘anonymised’ data, ‘de-identified’ data or data that has undergone ‘pseudonymisation’.^5^ However, the dynamic and multifaceted nature of big data, as well as the variety of data available, has increased the likelihood of privacy threats to data sets that are not readily identifiable. There is increased risk of (re)identification of individuals and/or a weakening of the security that data masking techniques appear to provide. Three kinds of disclosure risks may lead to the re-identification of an individual despite the masking or de-identification of identifiable data:

- *identity disclosure*—when data is successfully associated with person X;
- *attribute disclosure*—one such disclosure is made when person X is identified as belonging to a particular group, e.g. cancer registry, so there is membership disclosure; and
- *inferential disclosure*—when information about person X can be inferred with high confidence with released data (Templ 2017).

Disclosure risks can only ever be completely eliminated if data is not shared at all. Privacy models take into account the attributes of a dataset (as well as specific ‘attacker’ models) and specify the conditions that the data must satisfy in order for the disclosure risk to be minimised to an acceptable risk level^6^ (Soria-Comas and Domingo-Ferrer 2016). However, for the reasons given above, the anonymisation of data with the aid of privacy models is not perfect because these models were originally developed for static data sets rather than for use in the big data environment (Domingo-Ferrer and Soria-Comas 2016). There have been concerted efforts in recent years to identify what properties a privacy model should display to ensure that the privacy of data contributors is adequately protected while at the same time ensuring that data is not rendered useless as a result of privacy protection efforts. Although not an exhaustive list, three desirable properties that a privacy model for big data should display include:

- *data linkability*—i.e. the ability for anonymised data to remain relatively linkable so the value of the data is not significantly diminished;
- *composability*—this relates to the privacy guarantees that can be given when data from multiple sources (to which the same or different privacy models have been applied) is integrated into one data-rich source (i.e. fused); and
- *low computation*—this relates to algorithmic efficiency, i.e. the algorithm uses a low number of computational resources, such as time or space (Domingo-Ferrer and Soria-Comas 2016).

Despite such efforts, technical measures to protect individuals’ interests and rights, such as anonymisation, continue to be challenged or even rendered redundant with many big data initiatives (Domingo-Ferrer and Soria-Comas 2016).

### Diminishing Key Role of Informed Consent

A traditional and heavily relied on requirement for including individuals in research or other health-related activities has been informed consent. This standard is essentially an individuals’ agreement to assume the potential risk(s) involved in participating in the research or health activity. Heavy reliance on consent is becoming increasingly impracticable in the big data context because data might be linked and used within and across ecosystems that are far removed from the original source of information. While individuals might be re-contactable in some cases, it might still not be possible to inform them fully of the range of uses to which their data might be put by multiple users across countless ecosystems. The sheer size of participant involvement, the funding limitations, and the limited research timeframes imposed by funders also impose pragmatic limitations on re-contacting participants. In such circumstances, it is important to explore alternative ethically acceptable approaches and mechanisms which provide appropriate protections for individuals whose data may be used. This Framework takes into account these and other relevant and emerging issues.

### Aims of the Framework

The aim of this Framework is to examine the nature of the ethical issues raised by big data in health and research by identifying and bringing to the fore key underlying values and providing a step-by-step approach to thinking through the issues (Box 2).

**Box 2:** Purpose of the Framework

This Framework aims to 1. support decision-makers in identifying values relating to a range of big data uses, such as sharing, linkage, granting access to third parties 2. provide decision-makers with examples of a balancing approach to weighing up the relevant values when making decisions about big data; and 3. demonstrate how decision-makers can be more robust and transparent in their decision-making, thereby better equipping them to justify their decisions about the use and sharing of big data. This Framework does *not* aim to 1. provide a single set of standard issues or concepts relevant to *all* big data activities, as these may differ considerably; or 2. provide a single solution for specific issues that arise in big data activities.	

### Intended audience

The target audience of this Framework includes anyone who is accountable for big data in health and research. Specifically, the Framework is intended to be of relevance to:

1. Biomedical researchers, clinician-researchers, and data scientists;
2. Policymakers and those involved in governance of big data activities in health and research (including ethics committees and data access committees);
3. Data controllers with legal responsibilities for the safe and secure processing of personal data.

Beyond these three core groups, the Framework is also intended to be a helpful resource for academics and healthcare providers interested in thinking about and discussing ethical issues surrounding big data as well as patients, research participants, and lay people with an interest in this topic.

## Framework Structure

The Framework highlights key ethical values underlying a variety of big data activities. These values encompass nine *substantive* and seven *procedural* values that apply in big data activities.^7^ With a focus on these underlying values, the Framework provides ethical guidance on how key stakeholders in big data can think through issues to come to a decision in a number of domains where big data is used or shared. An important part of this process involves identifying and giving due consideration to all the relevant issues, identifying the relevant values, and weighing up values which appear to be in conflict with each other. Assisting with this process is a step-by-step guide, which articulates a structured decision-making process, and which is central to many decision-making frameworks. The value of this step-by-step approach is that it focuses decision-makers’ attention on the fact that a range of issues and values needs to be considered and prompts them to more thoroughly justify choices made, particularly where conflicting values are at play. The Framework is flexible enough to support decision-making across a diversity of big data activities.

The Framework is novel because it explores six key domains of big data in health and research in which underlying substantive and procedural values are examined in detail. These domains cover:

1. *Openness in big data and data repositories* (Xafis and Labude 2019)
2. *Precision medicine and big data* (Schaefer et al. 2019)
3. *Real-world data to generate evidence about healthcare interventions* (Lipworth 2019)
4. *AI-assisted decision-making in healthcare* (Lysaght et al. 2019)
5. *Big data and public-private partnerships in healthcare and research* (Ballantyne and Stewart 2019)
6. *Cross-sectoral big data* (Laurie 2019).

The articulation of issues specific to each domain enabled the identification of relevant values and subsequent analyses and balancing of competing values in a practical work-through fashion. It was not feasible to consider every possible issue pertinent to each domain. We therefore selected a subset of relevant issues and, for each issue, a subset of relevant values that need to be considered when engaging in moral reasoning to illustrate how the process works.

The domains have been designed to complement each other so that when read as a collective, they will cover a range of issues and articulate a number of values relevant to big data in health and research. In other words, while the domains can be read separately to explore some of the issues affecting a particular sector, there is benefit to be had from reading them together to get a broader view of the range of issues and values in operation across a range of data ecosystems.

The Framework sits within a context of broader issues that relate to and influence all decisions in big data, irrespective of the specific domain. These overarching ethical issues are *respect for persons*, *positive community expectations* known as ‘*social licence*’, and *vulnerabilities and power*.

## Three Overarching Ethical Issues

The section below provides a very brief account of issues that are central to the consideration of all domains in which big data is used. The section briefly discusses the concepts of ‘respect for persons’ and ‘social licence’ and describes the relationship between the two because this may not be readily evident. The examples help to elucidate the centrality of respect for persons and social licence in the big data context. The section also explores in depth the concept of vulnerability in the context of big data.

### Respect for Persons and Its Relationship to Social Licence

*Respect for persons* relates to one’s moral attitude towards others and the actions towards others that result from and exemplify this attitude. This moral attitude can entail both actions and omissions and can be displayed by individuals, groups, or institutions. We have not listed *respect for persons* as a specific value in this Framework. This is because it underpins and/or is intertwined with many of the values we have identified.

A thorough understanding of the concept of *respect for persons* requires us to be specific about what respect entails, who is entitled to respect, and what limits (if any) of respect might be justified. Historically, respect for persons has always been associated with acknowledging and respecting individuals’ autonomy (Lysaught 2004) but the concept of *respect for persons* is controversial, primarily because of debates about who counts as a ‘person’. We do not attempt to resolve these debates here but do want to clarify how we use the concept in the Framework. We endorse a broad conception of *respect for persons* that can accommodate a variety of cultural norms, including those which place less emphasis on individual autonomy and autonomous decision-making than is the norm in some cultures.

Understanding how exactly *respect for persons* manifests itself in the big data context and what it entails may, at first glance, appear elusive. *Respect for persons* is demonstrated in the moral stance or attitude we adopt towards individuals or groups and can be powerfully conveyed through communication. Likewise, a failure to communicate may be perceived as conveying a lack of *respect for persons*. We therefore need to examine communication in the context of big data further and link the concept of *respect for persons* to social licence.

Key to any relationship underpinned by respect is the establishment of a ‘conversation’ where participants have equal opportunity to engage with one another in a transparent and collaborative manner. The availability of reliable and digestible information on big data in health and research underlies any meaningful interaction that professionals, communities, and governments may hope to establish with publics.^8^ Transparent information on how big data is regulated, the protections in place for individuals whose data may be used, as well as the potential risks, the governance mechanisms adopted, clear accountability pathways, and clarity of the public benefits arising from such uses of health data are all essential elements of one side of the ‘conversation’ with publics. A further critical display of respect includes engaging with publics to understand and consider *their* views, concerns, and expectations in relation to uses of big data, including both aggregate and individual level health data. It is through such respectful interactions that public trust is built and ultimately secured, as a result of bodies/agencies being viewed as trustworthy.

The domain paper on *Big Data and Public-Private Partnerships in Healthcare and Research* (Ballantyne and Stewart 2019) discusses the destructive effects of deploying big data programmes without adequate clear information, consultation, or public engagement, even if the legal frameworks within which they are designed are sound (Carter et al. 2015; van Staa et al. 2016). To be truly respectful, such public interaction needs to take place not simply to ward off public backlash but as a matter of priority at the outset of new developments or programmes. Such was the approach adopted in the *Scottish Health Informatics Programme* (www.scot-ship.ac.uk/about.html), a Scotland-wide electronic patient records programme that has enjoyed great public support. The conversation needs to be ongoing and genuine in intent so as not to breach the public’s trust, as has been the case where public messaging lacks clarity and where public engagement has been insufficient.

Positive public expectations associated with the perceived legitimacy of activities that have broad societal impacts are referred to as ‘social licence’. We can find out whether a public is likely to accept or oppose data activities via research, public engagement activities such as citizens’ juries and focus groups, citizen responses to media releases, and social media such as Twitter. The expectations of publics regarding the acceptability of big data activities are heavily influenced by the level of engagement with publics and appropriate information dissemination (Hill et al. 2013).

In the previous section ‘Diminishing Key Role of Informed Consent’, we discussed the role of informed consent as the key mechanism for securing agreement for secondary uses of health data in research and other big data activities. Respecting persons entails not making generalised assumptions about social licence for the use of health data without consent. Equally, however, it entails not making generalised assumptions about social licence even where consent has been obtained from individuals.

*Social licence* depends on the specific context for a data activity (Xafis 2015), the partners involved, and local cultural norms. This is partly what makes cross-sectoral data sharing and public-private partnerships challenging. Therefore, beyond the broad dissemination of information and public engagement concerning big data, it may be that similar local efforts need to be made to ensure that uses of big data cohere with public expectations and values. This is a process central to any big data activity and needs to occur throughout the data activity, especially given the rapidly changing data ecosystem. Box 3 summarises key points to remember about respect for persons and social licence.

**Box 3:** Key points about *respect for persons* and *social licence*

1. *Respect for persons* is a moral attitude that individuals, groups, or institutions hold and display towards others. 2. *Social licence* relates to the positive public expectations associated with the perceived legitimacy of activities that have broad societal impacts and it also relates closely to trust, which, in turn, is enhanced via open, transparent communication. 3. The level of respect we hold towards others is often evident through interpersonal communication. 4. Showing respect towards publics in relation to the use of big data entails engaging in a variety of communicative exchanges to share information about big data activities and to receive input from publics. 5. Engaging with publics in such a way is a process that should be ongoing if public trust is to be promoted and achieved.	

### Vulnerabilities and Power in Big Data

A theme running through all of the domains in this Framework is that agents may be vulnerable to harms and/or wrongs that arise through the use of big data in health and research. In this section, we explore the ways in which activities using big data can create, exacerbate, or mitigate vulnerabilities. An important first step is to clarify what is meant by ‘vulnerability’.

#### The Nature of Vulnerability

In the most basic sense, vulnerability means susceptibility to harm or wrong, be that physical, social, or economic; it may be a characteristic of individuals or groups. It may be considered a characteristic of any human life, since we are all vulnerable to harms or wrongs of one sort or another. Yet this reflects neither how ‘vulnerability’ is used in everyday language, nor its moral importance: as Rogers has suggested, a wide definition of vulnerability ‘obscures rather than enables the identification of the context-specific needs of particular groups’ (Rogers et al. 2012). Meanwhile, Henk ten Have suggests that ‘[w]hat makes vulnerability problematic is the possibility of abuse and exploitation’ (ten Have 2016). This helps explain why the Declaration of Helsinki, which sets out the standard principles that govern medical research on humans, states that ‘[s]ome groups and individuals are *particularly* vulnerable and may have an *increased likelihood* of being wronged or of incurring additional harm’ (World Medical Association 2013).

For the sake of this document, we understand ‘vulnerability’ to mean an elevated susceptibility to systemic disadvantage, or a diminished opportunity for flourishing, arising from a physical, psychological, or social contingency. Correspondingly, the vulnerable are at an elevated risk of harm or wrong arising from such contingencies. It is important to keep in mind throughout that ‘vulnerability’ is likely to be context-specific, in that a person may be vulnerable in a certain way in situation *A*, but not in *B*, and vice versa. As Luna has argued, it may be less fruitful to think of a person as being vulnerable, than to think of them as being in a situation that renders them vulnerable; ‘[i]f the situation changes, the person may no longer be considered vulnerable’ (Luna 2009).

Given this, it is possible to identify a reasonably clear ‘family’ of more specific senses in which the word might be used.^9^At the most fundamental, *inherent vulnerability* is a feature of all human life: each of us is inherently vulnerable to illness, for example. However, as we have already indicated, to use the word in this way may be over-inclusive, and diminish its power to bring particular moral needs to light; and so it is useful to refine the concept so that it is sensitive to the contexts in which agents exist. To take an example relevant to this Framework, each of us is vulnerable to the (mis)use of data; this vulnerability is a function of the particular details of the information ‘ecosystem’ in which we live and our place within it, though, and so is situational rather than inherent. In addition, vulnerability may be more or less enduring; our vulnerability to the (mis)use of data may be magnified in some situations.

A person may be vulnerable to something, even if it never has any effect on their day-to-day life. For example, a participant in a pharmaceutical trial is vulnerable because the precise effects of the drug are unknown, but good regulation and monitoring will reduce the likelihood that the person is harmed. Likewise, anyone about whom data is gathered is vulnerable to the possibility of a data-breach, but that layer of vulnerability can be minimised through robust data-handling procedures. For some commentators, this speaks to a distinction between *occurrent* and *dispositional* vulnerability, where the former brings a risk of harm in the present, and the latter brings a similar risk in future. However, this distinction may not be all that useful, since ‘vulnerability’ implies susceptibility to harm or wrong; there is no timescale in this, and if a harm or wrong is present, then the object of our moral concern is no longer vulnerable: she is actually *being* harmed or wronged.^10^

Finally, vulnerability may be *pathogenic*. Pathogenic vulnerabilities ‘may be generated by a variety of sources, including morally dysfunctional or abusive interpersonal and social relationships and socio-political oppression or injustice’ (Rogers et al. 2012). For example, a patriarchal social structure can generate vulnerability. In another context, economic injustice may increase the vulnerability of certain groups to exploitative practices: for example, it is possible that the consent of would-be research participants is unreliable if participation represents their only realistic access to any medical treatment at all. In respect of big data, there may be a pathogenic aspect to vulnerability if an agent’s inability to control how information about him/her is used is related to the political or legal norms that apply to him/her.

Different kinds of vulnerability may often be found together, and vulnerability in one respect might be a factor in generating vulnerability in another under certain conditions. Vulnerability is also linked to a person’s relationship with other people and institutions and can therefore be analysed in terms of agents’ relative power—economic, political, or intellectual—over their own or others’ lives (Zion et al. 2000). Some relationships may reduce vulnerability; others may generate or exacerbate it. Perplexingly, vulnerabilities can be created or exacerbated by attempts to mitigate other vulnerabilities. Lange offers the example of a research protocol that excludes pregnant women, noting that

[w]hile the intent is to protect the woman and her fetus, the unintended effect is a lack of medications that have been adequately tested and approved for use during pregnancy. This lack in turn increases the vulnerability of all pregnant women (Lange et al. 2013; Dodds 2008).

Similar concerns might be raised in other contexts. Imagine that researchers are working on a treatment for a disease that is mainly found among the world’s poorest, and need to carry out trials involving them. While there are legitimate worries about exploitation, overplaying them may have the paradoxical effect of delaying research into the disease, thereby entrenching another vulnerability. To push the example further, research into rare diseases may be much easier if medical records and other relevant information from a range of sources can be collated; this is an archetypal use of big data. But such collation may bring the risk of de-anonymisation; the question to be addressed would be one of whether that risk is so great as to warrant abandoning the research: it might not be. In other words, it is not enough to say that we should be concerned by vulnerability, since vulnerability may take several forms and be difficult to avoid.

Vulnerability, then, is a multi-dimensional thing. A set of actions may increase vulnerability in some senses, while at the same time mitigating it in others. It may not be something that can be eliminated. What we can do, however, is be cognisant of its forms, and strive to attenuate it where possible. Where it is not possible to attenuate vulnerability, there will be a need to decide whether the research is justified, given the nature and scale of the harms and wrongs faced by the data subjects. There is unlikely to be a simple formula for making such assessments.

#### Vulnerability, Health Research, and Big Data

Control of information from health research that uses big data represents one of the clearest problems for mitigating vulnerability. Data is generated on anyone registered in a health system, and on anyone who has participated in research; it is also gathered in non-health settings—say, derived from internet browsing histories, from online purchases, or from personal fitness or sleep-monitoring devices. These databases might be accessed and linked to draw health-related inferences. Clearly, almost everyone is vulnerable to a breach of privacy arising from this; weak regulatory oversight will exacerbate that vulnerability. Moreover, bodies with a commercial interest in selling health-related goods and services may be able to use shared data to pressure people into purchasing them: susceptibility to such pressure is itself a kind of vulnerability to outside influence. This point is touched on in the Domain paper AI-assisted Decision-Making in Healthcare (Lysaght et al. 2019). Additionally, certain groups or their members may be vulnerable to unwanted interference by the government or other bodies that is informed by inferences from big data analysis. If the government in question is benign, the harms arising from this may be small, but not all governments are benign. The harms and wrongs arising from breaches of privacy or even simple lapses in its protection are further magnified if the inferences drawn from data are inaccurate.

It is also important to remember that big data research can generate significant benefits, and forswearing certain data sources can raise its own problems. Discovering a correlation between an illness and some hitherto unexpected feature of sufferers’ lives can help with diagnosis, and may be an important part of generating a treatment, thereby mitigating a particular vulnerability. An under-researched group may not be properly represented in databases, potentially exposing members to harm or, at best, meaning that they miss out on benefits—another manifestation of vulnerability; processing data from a range of sources may allow researchers to develop treatments for certain illnesses, or public health strategies to reduce their occurrence in the first place.

Thinking clearly about vulnerability in the context of big data will not mean that the problems will go away, and that is not what we intend here. As we have seen, vulnerability is inevitable and will be a feature of any social interaction. However, the key message here is that by being aware of how vulnerabilities might manifest, it ought to be easier to mitigate their undesirable effects.

#### Issues of Vulnerability in Big Data Research

In this section, we will look at three vulnerability-related issues raised by big data-based research. These three issues are not the only ones where vulnerability is important, but they ought to give a sense of the problems that researchers may face, and of strategies that may be adopted, and will therefore inform other examples.

1. The *big data divide*, which is a term describing the situation in which the benefits arising from the collection and use of big data are not evenly shared.
2. *Group harms*, which is a term that describes the possible harms to the collective interests of a community arising from the use and misuse of big data.
3. *Co-governance*, which is a consideration that may ameliorate some of the problems raised by concerns about the big data divide and the possibility of group harms and wrongs by ensuring that all stakeholders have a say in decision-making over how data are gathered, stored, and distributed.

**The Big Data Divide**

The big data divide marks a difference between ‘sorters and sortees’ (Andrejevic 2014): those who control, generate, or purchase access to large databases, and those who merely feature in them. The divide is potentially very wide—and possibly widening, because of the ubiquity of electronic devices: the bigger a person’s online presence, the more data will be generated; and the bigger the databank, the more power accrues to those who have access to the data. Mittelstadt and Floridi talk about ‘data subjects [being] in a disempowered state, faced with seemingly insurmountable barriers to understanding *who* holds *what* data about them, being used for *which* purposes’ (Mittelstadt and Floridi 2016). One concern is that those who are already in the most vulnerable social positions are likely to be among those most vulnerable to data use and misuse, yet also among those least able to do anything about it.

Uneven access to data (and to its benefits) is not necessarily unjust, but it might be unjust if that unevenness would not have arisen but for one party’s vulnerability, or if it results in vulnerabilities being exploited; it may not harm anyone, but it may prevent certain benefits accruing to those in need. Addressing the big data divide therefore requires that we engage with questions about justice, and about solidarity—particularly with those who are most vulnerable. Themes of justice and solidarity will recur throughout the Framework.

**Group Harms**

Vulnerability may be a characteristic of individuals or of groups. The everyday understanding of the word can accommodate this easily: it is not unusual to talk of cultures, communities, or the environment as vulnerable. Harms may accrue to individuals insofar as that they are members of a group, or to the group as a whole. However, harms to groups do not always mean that individual members are harmed. For example, if an Amazonian tribe is shrinking as its members assimilate into urban culture, we might say that it is vulnerable even if every individual member is flourishing. Generally, we should keep in mind that while it may be useful to talk of ‘vulnerable groups’, not every member of such groups will be vulnerable, vulnerable in the same way, or vulnerable only in the way that the group is; it would be a mistake to treat one as a perfect representative of the other (Luna 2009). In this light, the CIOMS *International Ethical Guidelines for Health-related Research Involving Humans* warn against treating ‘entire classes of individuals’ as vulnerable, though concede that ‘circumstances exist that require research ethics committees to pay special attention to research involving certain groups’ (Council for International Organizations of Medical Sciences (CIOMS) 2016).

There may be several ways in which considering group vulnerability may inform decision-making in health research. It might be that members of vulnerable groups are pressured into participation in research, and that the data generated are misused in a way that disadvantages that group. For example, the discovery that there is a link between a certain gene and a certain illness may impact on the lives of people who are identified as carrying that gene, irrespective of whether the illness ever manifests. This impact may be economic (relating to things like the cost of insurance), or socially stigmatising. Questions about who has the power to determine what happens to data are worth asking—questions that we shall also raise in respect of co-governance.

Another important point is that big data gives rise to a growing potential for re-identification of what was thought to be anonymised data, as discussed in a previous section on the Diminishing Key Role of Informed Consent. This may put individuals at an elevated risk of being harmed or wronged.

Equally, big data use might help ameliorate or avert group harms. Consider again the historical exclusion of pregnant women from medical trials, often for the sake of admirable concerns about the vulnerability both of the woman and of the embryo. US federal regulations for the protection of human research subjects stipulate that research involving pregnant women is permitted only if it ‘hold[s] out the prospect of direct benefit for the woman or the fetus; or, if there is no such prospect of benefit, the risk to the fetus is not greater than minimal and the purpose of the research is the development of important biomedical knowledge which cannot be obtained by any other means’ (Department of Health and Human Services 2017).

This has meant that there are gaps in our knowledge about how drugs affect around half the people using them due to differences between the male and female physiology and yet the drugs may get administered to women all the same, not least in the time before a woman knows that she is pregnant (Sinclair et al. 2016). Gathering real-world data on any sequelae may therefore be the only available method of learning about effectiveness and side effects, and therefore of ameliorating vulnerability (see for example (Lipworth 2019)). As noted above, similar considerations may be applied to research involving children, or on research into rare or ‘orphan’ diseases, for which sweeps of large datasets may provide otherwise unavailable insights into prevalence, aetiology, and possible treatment (Costa 2014; Pogue et al. 2018; Austin and Gadhia 2017). However, for some problems that need to be addressed with this notion, see (Halfmann et al. 2017).

One possible response is that governments should intervene on behalf of constituent groups for which they have responsibility, and pass laws to protect them from potential harms. Mexico, for example, has passed a law stipulating that genetic data is property of the government; the use and export of data, particularly when a patentable outcome is envisaged, therefore requires approval (Benjamin 2009; Séguin et al. 2008). Such legislation may be seen as an attempt to protect vulnerable groups from exploitation by large and wealthy biotech companies: a national government can shield minority groups. In this way, government and people can exercise co-governance over data, facilitate a way for groups to capitalise on their ‘own’ genetic resources, and reduce group harms, and tame commercial imperatives. On the other hand, legislation like Mexico’s may be seen as appropriating the rights of some groups to decide for themselves how to handle data derived from their genes. It may diminish, not bolster, the authority of peoples within the state.

There is no clear-cut position to be had on whether big data causes group harms, or how any such harms should be addressed. Its use might exploit and exacerbate certain group vulnerabilities, but might help us avoid others. It will therefore be important for data managers to confront the complexity of the issues surrounding big data use. It would also be important not to be irrationally optimistic about the benefits to be had; a precautionary approach may be desirable.

**Co-governance**

Co-governance implies ‘[s]haring and allocating roles, funds, responsibilities [and] powers’, and sharing in the formulation of the protocols for research programmes (Jones et al. 2001). In respect of research using big data, it means a system under which all stakeholders have the greatest possible say in how data about them is gathered, stored, and disseminated. This would help minimise the risks of, and arising from, violations of individuals’ privacy, group harms, and so on. Finally, co-governance also means taking seriously the arguable right of those biotech companies to have a say in how data is to be used and to a share of the benefits, since they will be investing the capital.

It is important to remember that co-governance is not a magic bullet: it will not make vulnerabilities, or the associated harms and wrongs, go away. One difficulty is that allowing *all* members of a group a say on how data are used will be impossible for any but the smallest groups, and it may be unclear what to do if members of the group disagree with each other. Appealing to the idea that a community may have representatives to speak on its behalf (Weijer 2006) simply changes the form of the problem: what does it mean to be representative, or *a* representative, of a group? What happens if there is a conflict of interests between members of the community and the community as a whole?

Co-governance cannot always reconcile competing moral imperatives, and simply appealing to co-governance will not tell us how to maintain due regard for data subjects’ privacy while making datasets available to researchers when that is desirable, granted that such maintenance is possible. Neither can it solve the problem of discoveries being made about participants without their ever knowing that a given question was being asked.

Co-governance may therefore be an ideal rather than a reality. Nevertheless, ensuring transparency and accountability in data use may ease some potential problems, and shows respect for stakeholders even when ‘full’ co-governance is not possible. Box 4 summarises key points to remember about vulnerability and power in big data.

**Box 4:** Key points about *vulnerability and power in big data*

1. Vulnerability takes several forms. 2. Vulnerability is often contextual; a person may be vulnerable in one situation but not another. 3. Using big data in health research provides a way to relieve some vulnerabilities but it might generate or exacerbate others. 4. Those handling big data should be aware of this, and consider ways in which possible harms and wrongs may be mitigated or avoided entirely.	

## Key Ethical Values

The Working Group identified 16 ethical values as being particularly important in the context of big data in health and research. It should be emphasised that this list of values is not exhaustive and that stakeholders may have other values or commitments that would be particularly important in certain contexts. This section provides an explanation of how the Working Group understands these values within the context of the Framework.

### Competing Values

Although all of the values referred to in the Framework may be easily satisfied in isolation, it will typically be impossible to simultaneously satisfy the demands implied by all the values. For example, data sharing might serve the value of public benefit but undermine privacy.

In cases where values are in tension with each other, decision-makers will need to judge which should take priority; the Framework cannot and does not include guidance on how to prioritise them. It is important to note that judging the best course of action will require more than comparing the length of the lists of values that would be satisfied or violated by a course of action. Rather, it is necessary to make a case for either compromising or privileging some values over others, and to put in place mechanisms to minimise the potential harms and wrongs associated with any chosen course of action.

### Substantive and Procedural Values

A broad distinction can be drawn between substantive and procedural values. *Substantive values* are those considerations that should be realised through the outcome of a decision. *Procedural values* are the values that guide the process of deliberation and decision-making itself.

Though substantive and procedural values are distinct, there is an important relationship between them in that procedural values may assist in realising certain substantive values. For example, maintaining the procedural value of *transparency* can help promote the substantive value of *justice* by allowing scrutiny from third parties who may be able to point out potential research discrepancies.

Procedural values are especially important in cases where we can expect reasonable people to disagree about which substantive values to prioritise. Current uses of big data are likely to generate such debate. It is therefore important that procedural values guide decision-making to ensure reasonable and defensible decisions are made in the face of a plurality of views. To illustrate what we mean, we offer the following example: given inescapable limits on resources, people might disagree about which healthcare needs should be prioritised; this would be a disagreement about substance. However, they might nevertheless agree on a fair rubric by which spending decisions should be taken and the deadlock broken; this would be an agreement about procedure.

We do not offer a specific formula for when or how to use or prioritise particular substantive or procedural values here. However, throughout the domain papers we offer many illustrative examples to show where and why values might clash, and how these tensions could be successfully navigated. There may be cases in which the distinction between procedural and substantive values comes very close or begins to blur. In such cases, the distinction is less important than it might be in others and decision-making should be focused on the discussion and consideration of the values themselves.

### The 16 Values

Listed in Tables 1 and 2 below are the substantive and procedural values identified as relevant to big data in health and research with their definitions.

**Table 1:** Substantive values relevant to big data contexts

Substantive value	Definition
Harm minimisation	*Harm minimisation* involves reducing the possibility of real or perceived harms (physical, economic, psychological, emotional, or reputational) to persons.
Integrity	*Integrity* refers to a feature or property of those acting in accordance with personal and/or accepted scientific and professional values and commitments.
Justice	*Justice* consists in treating individuals and groups fairly and with respect. This includes the fair distribution of benefits and burdens of data activities (collection, storage, use, linkage, and sharing) and attention to issues of equity.
Liberty/autonomy	*Liberty* and *autonomy* are very closely related concepts. For the purpose of this document, we define *liberty* as the state of not being coerced by physical, legal, or social pressure into action by some outside influence. *Autonomy* is defined as the capacity of a person or group to be self-determining.
Privacy^1^	For the purposes of this Framework, *privacy* refers to controlling access to information about persons. *Privacy* is valuable because the ability to control access to information about persons promotes certain core interests that we have as individuals and groups. These are wide-ranging but include identity interests and the promotion of human autonomous decision-making, as well as freedom from potential harms such as discrimination and stigmatisation that may arise from our data being disclosed. This control may be exercised directly by individuals to whom the data pertains, or by designated persons, such as data custodians whose decisions aim to promote those core individual and group interests.
Proportionality	*Proportionality* is a consideration in decision-making that requires that the means are necessary and appropriate in relation to the end that is being pursued, and being cognisant of the competing interests at hand.
Public benefit	*Public benefit* is the overall good that society as a whole receives from a given project. This includes consideration of effects on wellbeing, distribution, societal cohesion, human rights, and other sources of value to society. It may not be possible to measure these factors by the same standards, so some judgement and critical analysis will be required in determining what is publicly beneficial.
Solidarity	*Solidarity* is the commitment among persons with recognised morally relevant sameness or similarity to sharing costs and benefits for the good of a group, community, nation, or global population.
Stewardship	*Stewardship* reflects a relationship with things, such as data, to promote twin objectives of taking care of the object of attention as well as seeking actively to promote its value and utility. It involves guiding others with prudence and care across one or more endeavours—without which there is risk of impairment or harm—and with a view to collective betterment.

^1^Confidentiality should be considered alongside any privacy consideration, where relevant. The obligation to protect and promote the non-disclosure of information imparted in a relationship of trust lies at the core of the concept of confidentiality

**Table 2:** Procedural values relevant to big data contexts

Procedural value	Definition
Accountability	*Accountability* refers to the ability to scrutinise judgements, decisions and actions, and for decision-makers to be held responsible for their consequences.
Consistency	In the absence of relevant differences between two or more situations, *consistency* requires that the same standards be applied across them. While *consistency* in decision-making may be regarded as valuable in its own right, adherence to a practice of consistency may help actors to secure other values, such as fairness and trustworthiness.
Engagement	*Engagement* is the meaningful involvement of stakeholders in the design and conduct of the data activities. *Engagement* goes beyond the dissemination of information and requires that data activities have been influenced in some way by the views of stakeholders.
Reasonableness	*Reasonableness* means appealing to reasons and values that are widely recognised as relevant and fair.
Reflexivity	*Reflexivity* refers to the process of reflecting on and responding to the limitations and uncertainties embedded in knowledge, information, evidence, and data. This includes being alert to competing and conflicting personal, professional, and organisational interests and to the management of associated biases. Reflexive institutions revise or create new policies and systems that change institutional processes and prompt further reflection and response.
Transparency	*Transparency* is openness to public scrutiny of decision-making, processes, and actions. *Transparency* helps to demonstrate respect for persons and contributes to trustworthiness.
Trustworthiness	*Trustworthiness* is the property of being worthy of trust. It is a value that applies not only to individuals, organisations, governments, and institutions, but also to data, evidence, and systems. It can manifest procedurally as being transparent and truthful, reliable and consistent, or dependable.

### Process for Determining Key Values

The 16 substantive and procedural values listed in our Framework were adopted after a process of critical reflection by members of the SHAPES Working Group. The aim was to articulate a set of values that adequately capture the normative concepts that underpin ethical concerns surrounding big data in health and research. These values needed to be general enough to cut across multiple domains of big data use that are addressed in this Framework, but not so general as to blur together distinct spheres of concern.

Initially, a larger list of values was identified during a full-day face-to-face research meeting where detailed discussions were held. The SHAPES Working Group then merged values that were redundant, and set aside those insufficiently relevant to the issues raised by big data. The values were then classified as either substantive—referring to those that should be realised through the outcome of a decision, or procedural—referring to those which relate to ways we engage in human activities, including decision-making.

The group did not intend to settle considerable debate in the literature concerning the precise meaning of concepts like *justice* or *privacy*, but rather sought definitions that were concise, comprehensible to non-expert stakeholders, and reflective of the core ethical concerns raised by big data in health and research.

## Applying the Values: a Deliberative Balancing Approach to Decision-making

Within each of the six big data domains, we identified a set of relevant ethical issues and a subset of values that would be appealed to when deliberating about the issues. This is followed by a case study that illustrates how the values can be usefully deployed in order to assess what actions could/should be taken in a given context, and how that course of action can be justified.

While the case studies in the domain papers are not all identical in structure, they are all premised on the understanding that values inevitably compete and conflict, and that decision-makers need to judge which should take priority. This, as noted previously, requires more than comparing the lists of values that would be satisfied or violated by a course of action. Rather, it is necessary to provide justifications for either compromising or privileging one value over another, and to ensure that harms potentially arising from the chosen action are considered carefully and minimised to the greatest extent possible.

**Diagram 1:**
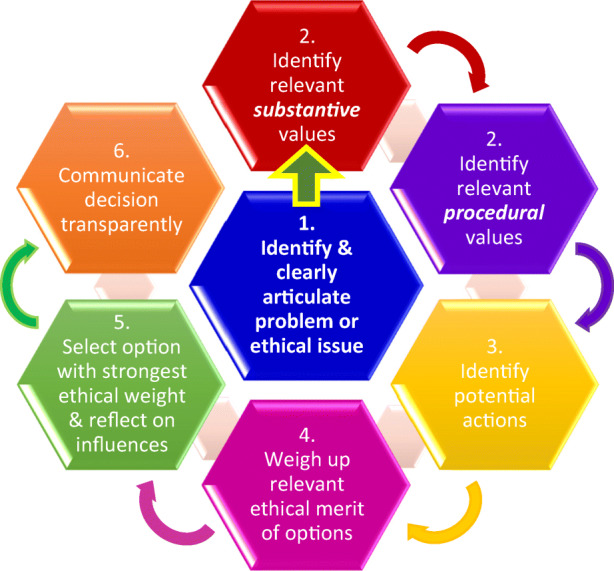
Deliberative balancing approach to decision-making in big data contexts

Broadly speaking, the case studies in the domain papers apply a process of reasoning that has been adapted from systematic, procedural approaches to ethical decision-making used by a variety of scholars and practitioners in healthcare ethics.^11^ While there are numerous step-by-step approaches depending on the area and the structure of the framework in question, some more extensive than others, we have adopted the following steps for the purposes of this Framework (represented in Diagram 1):

1. Identify and clearly articulate the presumptive ethical issue or problem at hand
2. Identify the relevant values pertinent to the issue or problem (from the list of 16 key ethical values, noting, as previously stated, that this may not be an exhaustive list). This is a two-step process as *procedural* and *substantive* values need to be identified (see Diagram 1)
3. Identify potential actions (including consideration of policies, legal issues) that could be taken in response
4. In light of the values and context, weigh up the relative ethical merit of the different options
5. Select the option that has the strongest ethical weight attached to it and reflect on how your personal or the group’s position and interests have influenced the decision, noting that the decision may require further consideration
6. Communicate the decision transparently to all stakeholders.

We have previously indicated that overarching considerations include our moral attitude towards persons and groups (*respect for persons*), the need to make decisions that cohere with community expectations (*social licence*), and the need to ensure that research or activities using big data mitigate vulnerabilities rather than creating or exacerbating them. Considerations of *social licence* and efforts to alleviate and not exacerbate vulnerabilities also work towards promoting *respect for persons* and groups so we see that the moral attitude we have towards others is central to the whole decision-making process.

These three overarching considerations assist in identifying some of the values we need to consider in each case but there is likely to be an iterative process in steps 1, 2, and 3 in order to fully identify all the relevant issues and values. Also assisting in identifying the relevant values is the specific issue/problem and the context within which it arises. When we begin to consider some possible solutions, it is likely that additional values will become obvious and will lead us to continue to consider the specific solution, amend it, or discard it, as a result of the deliberative process.

The resolution of conflicts between values in step 4 is arguably the most difficult aspect of any ethical decision-making framework and it is difficult to articulate a single process for balancing conflicting values, particularly outside a specific context. *Deliberative balancing* is the process we engage in when trying to determine and justify which value carries greater weight than another relevant value in a particular case (Demarco and Ford 2006). The justifications and reasoning provided for considering one value to hold greater importance than another help promote and further moral debate and provide a clear basis on which decisions have been reached (Demarco and Ford 2006).

Robustly justified conclusions result from the use of such step-by-step decision-making processes, which make it less likely that stakeholders will overlook relevant values and considerations. This step-by-step process also has the advantage of being able to proceed with pragmatic analysis and discussion concerning ethical issues in big data without becoming bogged down in theoretical disputes. As previously noted, key requirements in this deliberative balancing and weighing process are deep insight, reflection, sound judgement, and acknowledgment and tolerance of uncertainty, all features of personal and general wisdom.

This structured step-by-step process eschews any particular approach to determining the best course of action, as may be found in utilitarian, libertarian, or human rights theories. While such theories may have the advantage of being able to produce definite answers with less critical reflection, they are each contentious. Ethical decision-making frameworks, instead, rely on values with some degree of commonality between different systematic theories (Beauchamp and Childress 2013). This has the procedural advantage of being more likely to be acceptable among stakeholders with a variety of background ethical commitments.

Taking the values, the broader overarching considerations, and the step-by-step decision-making process together, this Framework provides decision-makers with a means to engage explicitly with what is at stake in a given big data context; it provides a common language with which to interact with other decision-makers and stakeholders; it offers a process for thinking through specific decisions relative to the values and, importantly, for justifying final outcomes, and thereby supporting more robust ethical decision-making in the realm of big data.

Explicit articulation of the steps above is exemplified in the first of the domain papers (Xafis and Labude 2019) presented in this Special Issue. In the remaining domain papers, the issues are worked through using this approach but the steps are not always as overtly articulated. As discussed earlier, the present Framework is meant to assist stakeholders in making judgements and determinations for themselves, in a systematic way irrespective of their worldview. As previously noted, the domain papers achieve this by outlining the issues and values in the context of the particular domain and using specific cases and examples to work through the identified issues and values in a more concrete way.

## Feedback Cycles

This project and the formation of the Working Group was initiated by the *Science, Health and Policy-relevant Ethics in Singapore* (SHAPES) *Initiative*, Centre for Biomedical Ethics (CBmE), National University of Singapore (NUS). The SHAPES conference ‘*Ethics of Big Data in Health and Research’* was held on 30 Nov–1 Dec 2018 and was supported by the *Clinical Ethics Network + Research Ethics Support Programme*, CBmE, NUS.

This conference provided the opportunity for the first round of expert feedback. SHAPES introduced an innovative feedback loop at the conference, which was attended by experts in the field, clinician researchers, and government officials with an interest in big data. Following each domain presentation and discussion, attendees were asked to provide written feedback in relation to key issues the respective domain should discuss or any points they felt were important to note. This detailed feedback about the domains and the Framework as a whole was subsequently incorporated into the draft Framework document and the individual domain papers.

The draft Framework and individual domains were then reviewed by the whole SHAPES Working Group and were subsequently sent to experts from a variety of specialty fields for further comment (Table 3). The SHAPES Working Group greatly appreciates the thoughtful feedback provided and acknowledges that it contributed to an improved articulation of the Framework and domain papers.

**Table 3:** Expert feedback and commentary on the Framework

Reviewer	Affiliation
Dr Florencia Luna	Facultad Latinoamericana de Ciencias Sociales (Latin American School of Social Sciences), Argentina
Professor Mark Taylor	Deputy Director of HeLEX@Melbourne, University of Melbourne, Australia
Professor Patrick Tan	Director, Duke-NUS Genome Biology Facility, Duke-NUS Medical School, Singapore
Assistant Professor SIM Xueling	Saw Swee Hock School of Public Health, National University of Singapore, Singapore
Dr Nayha Sethi	Chancellor’s Fellow, Mason Institute, University of Edinburgh, UK
Dr Sarah Chan	Reader/Chancellor’s Fellow, Usher Institute of Population Health Sciences and Informatics, University of Edinburgh, UK
Professor Kenneth Goodman	Director, Institute for Bioethics and Health Policy; School of Medicine, University of Miami, USA

Following the expert feedback cycle, the SHAPES Working Group and the SHAPES team incorporated the feedback into the Framework, which was further reviewed by the whole SHAPES Working Group.

## Conclusion

The Framework presented in this paper identified 16 substantive and procedural values the SHAPES Working Group deemed relevant to numerous big data domains. While not exhaustive, the articulation of these values has the potential to elucidate important considerations in big data research and health activities. The Framework also highlighted three general issues that cut across all decision-making in big data contexts: *respect for persons*, *social licence*, and *vulnerability and power*. We presented a step-by-step deliberative process and clarified how relevant values can be identified. While decision-making frameworks cannot provide definitive guidance on how to balance conflicting values, this Framework points to issues which contribute to such balancing and articulates how a careful deliberative process can offer more robust justifications for decisions made in the intricate big data landscape. Examples of how users can work through a number of issues in different big data domains are provided in the individual domain papers which form part of this Framework.

**Table:** 

SHAPES Working Group	
Co-chairs:	
Associate Professor Tai E Shyong	Division of Endocrinology, National University Hospital and Saw Swee Hock School of Public Health, National University of Singapore
Professor Graeme Laurie	School of Law and JK Mason Institute for Medicine, Life Sciences and the Law, University of Edinburgh
Members in alphabetical order:	
Associate Professor Angela Ballantyne	Department of Primary Health Care & General Practice, University of Otago
Dr. Iain Brassington	Centre for Social Ethics and Policy, School of Law, University of Manchester
Mr. Markus Labude	Centre for Biomedical Ethics, National University of Singapore
Associate Professor Hannah Yeefen Lim	Division of Business Law, College of Business, Nanyang Technological University
Associate Professor Wendy Lipworth	Sydney Health Ethics, The University of Sydney
Assistant Professor Tamra Lysaght	Centre for Biomedical Ethics, National University of Singapore
Dr. Owen Schaefer	Centre for Biomedical Ethics, National University of Singapore
Professor Cameron Stewart	Sydney Law School, The University of Sydney
Associate Professor Shirley Sun Hsiao-Li	School of Social Sciences, College of Humanities, Arts, & Social Sciences Nanyang Technological University
Dr. Vicki Xafis	Centre for Biomedical Ethics, National University of Singapore

## Footnotes

1 However, decisions and actions are justified as part of the deliberative decision-making process.

2 A framework may, however, be guided by overarching considerations such as the need to respect persons, to take account of community expectations, and to consider issues of vulnerability which can arise in uses of big data, as is the case in this Framework.

3 “In general, anonymisation refers to the process of removing identifying information such that the remaining data cannot be used to identify any particular individual…Data would not be considered anonymised if there is a serious possibility that an individual could be re-identified, taking into consideration both: (a) the data itself, or the data combined with other information to which the organisation has or is likely to have access and (b) the measures and safeguards (or lack thereof) implemented by the organisation to mitigate the risk of identification.” (Personal Data Protection Commission Singapore 2018)

4 Information “that may reasonably be expected to identify an individual, alone or in combination with other data.” (Global Alliance for Genomics and Health 2015)

5 For example, in the US Health Insurance Portability and Accountability Act (2004) Security Rule; the EU General Data Protection Regulation (2016/679); and Singapore’s Personal Data Protection Act (2012).

6 As one of the experts we consulted pointed out, even if there is no re-identification risk present, individuals whose data is used in a non-identifiable manner (as well as others who share the same group membership) may nevertheless be impacted adversely by its use, as a result of decisions made based on the use of the data (e.g. policy decisions).

7 We have selected the values we considered most pertinent to big data but other values may also be relevant depending on the specific context within which ethical issues are being considered. There are also aspects of decision-making, such as efficiency, economy, and practicability, which although significant considerations in decision-making, are not considered to be ethical values and have not been discussed here. We acknowledge, however, that they should always be considered in such decision-making.

8 A way in which the Framework uses ‘public’ is as a noun, where it refers to the community of citizens. This sense of public is often invoked as a target audience for public engagement by policy makers. However, relying on this singular understanding of ‘the public’ has its drawbacks: it suggests that there is a representative public with internally consistent views about and attitudes towards a subject matter. A more nuanced picture may instead invoke the notion of multiple ‘publics’, with diverging levels of background knowledge and prior levels of engagement on the subject matter.

9 Despite some differences, this taxonomy is informed by (Mackenzie et al. 2014).

10 Our thanks are owed to one of the peer reviewers for making this useful point.

11 See, for example, WRHA Ethics Services 2015, Kerridge et al. 2013, and Velasquez et al. 2015.
